# ALBI score combined with FIB-4 index to predict post-hepatectomy liver failure in patients with hepatocellular carcinoma

**DOI:** 10.1038/s41598-024-58205-5

**Published:** 2024-04-05

**Authors:** Yi-Bo Tian, Hong Niu, Feng Xu, Peng-Wei Shang-Guan, Wei-Wei Song

**Affiliations:** 1 Department of Hepatobiliary Surgery, Jincheng People’s Hospital, Jincheng, 048026 Shanxi Province China; 2 Department of Emergency, Jincheng General Hospital, Jincheng, 048000 Shanxi Province China; 3 Department of Gastroenterology, Jincheng General Hospital, Jincheng, 048000 Shanxi Province China; 4 Department of General Surgery, Jincheng General Hospital, Jincheng, 048000 Shanxi Province China; 5 Department of Medical Quality Control, Jincheng General Hospital, Jincheng, 048000 Shanxi Province China

**Keywords:** Hepatocellular carcinoma, Hepatectomy, Post-hepatectomy liver failure, Albumin-bilirubin (ALBI) score, Fibrosis-4 (FIB-4) index, Surgical oncology, Hepatocellular carcinoma, Liver cancer

## Abstract

Post-hepatectomy liver failure (PHLF) is a potentially life-threatening complication following liver resection. Hepatocellular carcinoma (HCC) often occurs in patients with chronic liver disease, which increases the risk of PHLF. This study aimed to investigate the ability of the combination of liver function and fibrosis markers (ALBI score and FIB-4 index) to predict PHLF in patients with HCC. Patients who underwent hepatectomy for HCC between August 2012 and September 2022 were considered for inclusion. Multivariable logistic regression analysis was used to identify factors associated with PHLF, and ALBI score and FIB-4 index were combined based on their regression coefficients. The performance of the combined ALBI-FIB4 score in predicting PHLF and postoperative mortality was compared with Child–Pugh score, MELD score, ALBI score, and FIB-4 index. A total of 215 patients were enrolled in this study. PHLF occurred in 35 patients (16.3%). The incidence of severe PHLF (grade B and grade C PHLF) was 9.3%. Postoperative 90‐d mortality was 2.8%. ALBI score, FIB-4 index, prothrombin time, and extent of liver resection were identified as independent factors for predicting PHLF. The AUC of the ALBI-FIB4 score in predicting PHLF was 0.783(95%*CI*: 0.694–0.872), higher than other models. The ALBI-FIB4 score could divide patients into two risk groups based on a cut-off value of − 1.82. High-risk patients had a high incidence of PHLF of 39.1%, while PHLF just occurred in 6.6% of low-risk patients. Similarly, the AUCs of the ALBI-FIB4 score in predicting severe PHLF and postoperative 90-d mortality were also higher than other models. Preoperative ALBI-FIB4 score showed good performance in predicting PHLF and postoperative mortality in patients undergoing hepatectomy for HCC, superior to the currently commonly used liver function and fibrosis scoring systems.

## Introduction

Hepatocellular carcinoma is the sixth most prevalent cancer across the world and it is also ranked as the third most frequent cause of cancer-related deaths worldwide^1^. Hepatectomy is currently considered the primary form of curative treatment for HCC^2–4^. Compared to palliative treatments, hepatectomy could offer a better chance of prolonged survival for patients with HCC^5,6^. Advances in liver surgery and perioperative management has resulted in a noteworthy improvement in the safety of hepatectomy and a reduction in postoperative mortality^7^. However, post-hepatectomy liver failure (PHLF) remains a potentially life-threatening complication following liver resection and the primary cause of postoperative mortality^8,9^. Additionally, HCC often occurs in patients with chronic liver disease, which increases the risk of PHLF and perioperative death^3,8^. Accordingly, precise estimation of the risk of PHLF is crucial to enhance the safety of patients with HCC undergoing hepatectomy.

In general, predicting the risk of PHLF is primarily dependent on the evaluation of patients’ liver function reserve^2,3,8^. The Child–Pugh score and model for end-stage liver disease (MELD) score are the two most broadly used models for assessing liver function in patients with HCC^3,4,10,11^. The Child–Pugh score was initially designed to evaluate the prognosis of patients with portal hypertension receiving surgery for oesophageal variceal bleeding^10^. MELD was developed to predict survival in patients after transjugular intrahepatic portosystemic shunts (TIPS)^12^. Both the two models were not originally conceived to evaluate liver function reverse in patients with HCC. Previous studies have also shown that their predictive value in PHLF is not ideal^13,14^. The albumin-bilirubin (ALBI) score, proposed by Johnson *PJ *et al*.* in^15^, is based solely on the measurement of serum albumin and bilirubin level, which has been proved to be a more objective and accurate model for evaluating liver function in HCC patients^15,16^. Additionally, compared to the Child–Pugh score and MELD score, the ALBI score also showed more powerful ability in predicting PHLF^13,17^.

The majority of patients with HCC have a background of chronic liver disease, such as chronic hepatitis B virus (HBV) or hepatitis C virus (HCV) infection, nonalcoholic fatty liver disease (NAFLD), and alcoholic steatohepatitis^3^. Their degree of liver fibrosis is also associated with PHLF. Fibrosis-4 (FIB-4) index, calculated from three biochemical indices (aspartate aminotransferase (AST), alanine aminotransferase (ALT), and platelet count) and age, is a noninvasive marker for evaluating liver fibrosis and could provide accurate prediction for decompensation^18,19^. This study aimed to investigate the ability of the combination of liver function and fibrosis markers (ALBI score and FIB-4 index) to predict PHLF.

### Patients and methods

The study was approved by the Ethics Committee of the Jincheng General Hospital (Approval number: 2023060501) and carried out in compliance with the Helsinki Declaration. All patients provided written informed consent for the use of their data for scientific research.

### Patients

Patients who underwent hepatectomy for HCC between August 2012 and September 2022 at the Jincheng General Hospital were considered for inclusion. The inclusion criteria were the following: (1) hepatectomy as initial treatment for HCC; (2) the diagnosis of HCC confirmed by postoperative pathology. Patients were excluded if they (1) had obstructive jaundice before surgery; (2) received other preoperative treatments for HCC; (3) had cardiopulmonary dysfunction before surgery.

### Definitions

The definition of clinically significant portal hypertension (CSPH) was the presence of gastroesophageal varices or enlargement of the spleen (measuring more than 12 cm in diameter) along with reduced platelet count (less than 100 × 10^9^/L)^20^. Major liver resection refers to the surgical procedure that involves the removal of three or more hepatic segments from the liver^21^. According to the International Study Group of Liver Surgery (ISGLS) criteria, PHLF was defined as the simultaneously increased total bilirubin level and international normalized ratio (INR) on or after 5 days following the liver surgery^9^. Patients who have PHLF grade A do not need any specific treatment. However, those with grade B need some non-invasive therapies, such as administration of daily diuretics and infusion of fresh albumin or frozen plasma. Those with grade C require invasive procedures like hemodialysis, extracorporeal liver support, and mechanical ventilation. PHLF grades B and grade C were considered severe PHLF. Postoperative mortality was defined as the occurrence of death during the 90-day period following the surgical procedure. The staging of HCC was based on the Barcelona Clinical Liver Cancer (BCLC) staging system^2^.

### Hepatectomy

Prior to the surgical procedure, patients were required to undergo routine blood tests including liver and kidney function, blood cell analysis, coagulation function, HBV and HCV immunology, as well as alpha-fetoprotein (AFP). Patients detected with HBV infection were required to receive antiviral treatment (such as entecavir or tenofovir) for about 1 week before surgery. Contrast-enhanced CT or MRI scan was utilized to assess the morphology of the tumor and whether it was resectable. Preoperative liver function was evaluated using the Child–Pugh Score. Patients selected for hepatectomy had to meet the following criteria^22^: (1) good liver function (Child–Pugh grade A or B); (2) good performance status (Eastern Cooperative Oncology Group performance score of 0–2); (3) sufficient remaining liver volume (at least 40% for patients with liver cirrhosis or at least 30% for those without liver cirrhosis); (4) no extra-hepatic metastasis. Liver and kidney function, blood cell analysis and coagulation function test were routinely performed at 1, 3 and 5 days after operation.

### Calculations of the ALBI, Child–Pugh, MELD score and FIB-4 index

The ALBI score was calculated using the following formula: 0.66 × log_10_(bilirubin, μmol/L) − 0.085(albumin, g/L)^15^. The calculation of Child–Pugh score was determined according to previous report, and patients were stratified into 3 grades: grade A (5‐6 points), grade B (7–9 points), and grade C (10–15 points)^23^. The computed formula for the MELD score was 3.8 × log_e_ (bilirubin, mg/dL) + 11.2 × log_e_ (INR) + 9.6 × log_e_ (creatinine, mg/dL) + 6.4^24^. The FIB-4 index was calculated as follows: age [years] × AST [U/L])/(platelet count [10^9^/L] × ALT[U/L]^1/2^)^18^.

### Statistical analyses

Continuous data were described using median with interquartile range (IQR). Categorical data were expressed as frequency (percentage), and analyzed using *χ*^*2*^ test or Fisher’s exact test. Multivariate logistic regression analysis using a backward stepwise selection method was conducted to determine independent factors predicting PHLF. The area under the receiver operating characteristic (ROC) curve (AUC) was calculated to evaluate the ability of models in predicting PHLF and mortality. And the DeLong test was used to compare the AUCs for different models. SPSS version 23.0 software was used for statistical analysis, and *P* < 0.05 was considered statistically significant.

## Results

A total of 247 patients with HCC undergoing hepatectomy in the study interval were considered for inclusion. Of these, 32 patients (13.0%) were excluded because the following reasons: 11 (4.5%) had obstructive jaundice before surgery; 15 (6.1%) received anti-tumor treatments prior to hepatectomy; 6 (2.4%) had cardiopulmonary dysfunction at baseline. The remaining 215 patients were enrolled in this study.

### Characteristics of patients

Preoperative and intraoperative data of the included 215 patients with HCC are described in Table 1. The median age of the patients was 55 years, and 85.6% were male. Nearly 80% of patients had HBV infection, and cirrhosis accounted for approximately 70%. The vast majority of the patients (95.8%) had a Child–Pugh grade A liver function, and only 4.2% had a liver function of Child–Pugh grade B. The median (IQR) MELD score, ALBI score, and FIB-4 index were 7.9 (7.1–8.9), − 2.91 (− 3.19 to − 2.63), and 2.03 (1.43–3.40), respectively. Early staged (BCLC-0 and -A) HCC accounted for 62.8%, and major liver resection performed in 22.8% of patients.

**Table 1:** Baseline characteristics.

Variable	Data
Age (years)	55 (45–63)
Gender	
Male	184 (85.6)
Female	31 (14.4)
Positive HBsAg	167 (77.7)
Positive anti-HCV	6 (2.8)
Cirrhosis	148 (68.8)
Platelet count (10^9^/L)	164 (122–214)
Albumin (g/L)	42.9 (39.2–45.3)
Total bilirubin (μmol/L)	12.4 (8.8–16.2)
AST (U/L)	36 (26–51)
Creatinine (μmol/L)	79 (67–89)
Prothrombin time (sec)	13.0 (12.3–14.0)
AFP (ng/mL)	
< 400	137 (63.7)
≥ 400	78 (36.3)
CSPH	41 (19.1)
Child–Pugh grade	
A	206 (95.8)
B	9 (4.2)
MELD score	7.9 (7.1–8.9)
ALBI score	− 2.91 (− 3.19 to − 2.63)
FIB-4 index	2.03 (1.43–3.40)
Tumor size (cm)	6.0 (3.8–9.0)
Tumor number	
Single	175 (81.4)
Multiple	40 (18.6)
BCLC stage	
0 and A	135 (62.8)
B and C	80 (37.2)
Extent of resection	
Minor	166 (77.2)
Major	49 (22.8)
Intraoperative blood loss (mL)	
> 400	145 (67.4)
≤ 400	70 (32.6)
Intraoperative blood transfusion	35 (16.3)

Values were shown as n (%), or median (interquartile range).

*AFP* alphafoeto‐protein, *ALBI* albumin-bilirubin, *AST* aspartate aminotransferase, *BCLC* barcelona clinic liver cancer, *CSPH* clinically significant portal hypertension, *FIB-4* fibrosis-4, *HBsAg* Hepatitis B surface antigen, *HCV* Hepatitis C virus, *MELD* model for end-stage liver disease.

### Incidence of PHLF and mortality

A total of 35 patients (16.3%) developed PHLF, including 15 (7.0%) of grade A PHLF, 15 (7.0%) of grade B PHLF, and 5 (2.3%) of grade C PHLF. The incidence of severe PHLF (grade B and grade C PHLF) was 9.3%. Postoperative 90‐d mortality was 2.8% (6 patients). All the 6 patients died of PHLF.

### Uni- and multi-variable analyses to identify factors associated with PHLF

In the univariable analyses, preoperative variables including platelet count, albumin, total bilirubin, AST, prothrombin time, CSPH, ALBI score, FIB-4 index, and extent of liver resection were determined to be associated with PHLF (Table 2). In the multivariable analyses, ALBI score (odds ratio [OR] 2.457, 95%*CI*:1.012–5.970; *P* = 0.047), FIB-4 index (OR 1.215, 95%*CI*: 1.043–1.415; *P* = 0.012), prothrombin time (OR 1.477, 95%*CI*: 1.069–2.042; *P* = 0.018), and extent of liver resection (major resection: OR 2.433, 95%*CI*: 1.007–5.879; *P* = 0.048) were identified as independent factors for predicting PHLF (Table 2). To avoid collinearity, platelet count, albumin, total bilirubin, and AST were not enrolled into the multivariable analyses.

**Table 2:** Uni- and multi-variable logistic regression analyses to identify factors associated with PHLF based on preoperative data.

Variable	Univariable regression		Multivariable regression^a^	
	OR (95% CI)	*P*	OR (95% CI)	*P*
Age (years)	1.012 (0.981–1.044)	0.459		
Male	0.316 (0.072–1.388)	0.127	-	-
Positive HBsAg	0.548 (0.200–1.500)	0.242	-	-
Positive anti-HCV	2.667 (0.469–15.157)	0.269	-	-
Platelet count (109/L)	0.988 (0.982–0.995)	< 0.001		
Albumin (g/L)	0.891 (0.837–0.948)	< 0.001		
Total bilirubin (μmol/L)	1.092 (1.034–1.153)	0.002		
AST (U/L)	1.011 (1.003–1.019)	0.005		
Creatinine (μmol/L)	1.000 (0.990–1.011)	0.979	-	-
Prothrombin time (sec)	1.762 (1.315–2.360)	< 0.001	1.477 (1.069–2.042)	0.018
CSPH	5.221 (2.375–11.479)	< 0.001	1.979 (0.698–5.615)	0.199
ALBI score	4.728 (2.271–9.845)	< 0.001	2.457 (1.012–5.970)	0.047
FIB-4 index	1.304 (1.151–1.478)	< 0.001	1.215 (1.043–1.415)	0.012
Tumor size (cm)	1.061 (0.975–1.154)	0.172	-	-
Tumor number	0.888 (0.342–2.309)	0.808	-	-
Major resection	2.364 (1.087–5.140)	0.030	2.433 (1.007–5.879)	0.048

*ALBI* albumin-bilirubin, *AST* aspartate aminotransferase, *CSPH* clinically significant portal hypertension, *FIB-4* fibrosis-4, *HBsAg* Hepatitis B surface antigen, *HCV* Hepatitis C virus, *MELD* model for end-stage liver disease, *PHLF* post-hepatectomy liver failure.

^a^To avoid collinearity, platelet count, albumin, total bilirubin, and AST were not enrolled into the multivariable regression model.

In the multivariable logistic regression model, the regression coefficients for ALBI score and FIB-4 index were 0.899 and 0.195, respectively. Hence, the new ALBI-FIB4 score was calculated as follows: 0.899 × ALBI score + 0.195 × FIB4 index. The median (IQR) of ALBI-FIB4 score was − 2.12 (− 2.51 to − 1.72).

### Performance of ALBI-FIB4 score and other models in predicting PHLF

**Figure 1:**
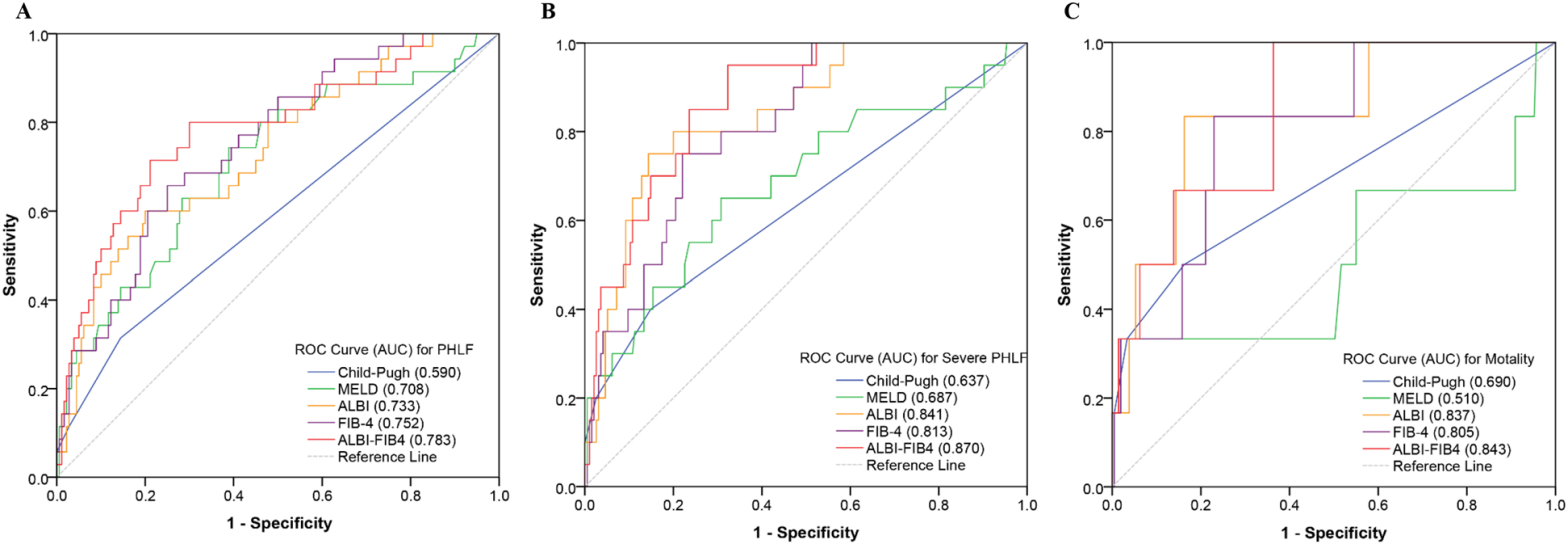
The ROC curves of the models for predicting PHLF (**A**), severe PHLF (**B**), and postoperative 90 days mortality (**C**).

**Figure 2:**
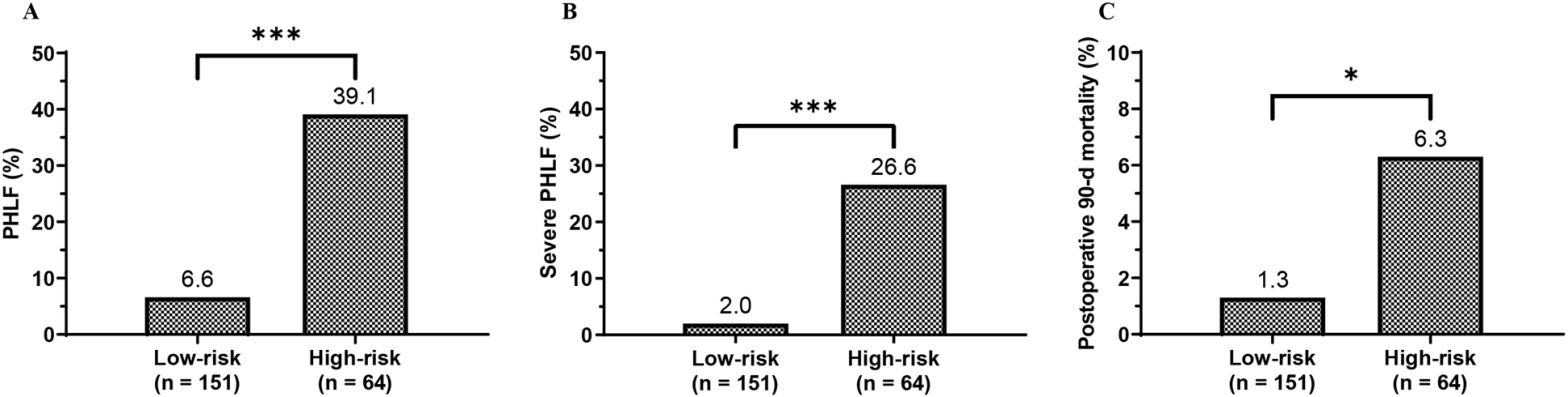
Comparison of incidences of PHLF (**A**), severe PHLF (**B**), and postoperative 90 days mortality (**C**) between patients with ALBI-FIB4 score ≤ − 1.82 (low-risk) and > − 1.82 (high-risk).

In the ROC analyses, the ALBI-FIB4 score showed a greater ability in predicting PHLF compared with other models (Fig. 1A and Table 3). The AUC of the ALBI-FIB4 score (0.783, 95%*CI*: 0.694–0.872) in predicting PHLF was significantly higher than the Child–Pugh score (0.590, 95%*CI*: 0.479–0.700; *P* = 0.001), also higher than the MELD score (0.708, 95%*CI*: 0.609–0.806), ALBI score (0.733, 95%*CI*: 0.641–0.825), and FIB-4 index (0.752, 95%*CI*: 0.669–0.835), although these differences were not statistically significant based on the DeLong test (all *P* > 0.05). The cut-off of the ALBI-FIB4 score for predicting PHLF was − 1.82, with a sensitivity of 71.4% and a specificity of 78.9%. Patients with ALBI-FIB4 score > –1.82 were considered high-risk (n = 64 (29.8%)), while those with ALBI-FIB4 score ≤ − 1.82 were considered low-risk (n = 151 (70.2%)). The incidence of PHLF in the high-risk group was 39.1%, significantly higher than the 6.6% in the low-risk (*P* < 0.001; Fig. 2A).

**Table 3:** Performance of models in predicting PHLF, severe PHLF, and mortality.

Models	PHLF		Severe PHLF		90 days mortality	
	AUC	95%*CI*	AUC	95%*CI*	AUC	95%*CI*
Child–Pugh score	0.590	0.479–0.700	0.637	0.492–0.782	0.690	0.430–0.949
MELD score	0.708	0.609–0.806	0.687	0.551–0.823	0.510	0.206–0.814
ALBI score	0.733	0.641–0.825	0.841	0.757–0.926	0.837	0.678–0.996
FIB-4 index	0.752	0.669–0.835	0.813	0.731–0.894	0.805	0.657–0.953
ALBI-FIB4	0.783	0.694–0.872	0.870	0.803–0.936	0.843	0.716–0.969

*ALBI* albumin-bilirubin, *FIB-4* fibrosis-4, *MELD* model for end-stage liver disease, *PHLF* post-hepatectomy liver failure.

The AUC of the ALBI-FIB4 score in predicting severe PHLF was 0.870 (95%*CI*: 0.803–0.936), also higher than other models (Fig. 1B and Table 3). The incidence of severe PHLF was 26.6% in the high-risk group, while only 2.0% in the low-risk group (*P* < 0.001; Fig. 2B).

### Performance of ALBI-FIB4 score and other models in predicting mortality

The ALBI-FIB4 score also showed a good ability in predicting postoperative 90 days mortality. The AUC of the ALBI-FIB4 score in predicting postoperative 90 days mortality was 0.843 (95%*CI*: 0.716–0.969), higher than the Child–Pugh score (0.690, 95%*CI*: 0.430–0.949), MELD score (0.510, 95%*CI*: 0.206–0.814), ALBI score (0.837, 95%*CI*: 0.678–0.996), and FIB-4 index (0.805, 95%*CI*: 0.657–0.953) (Fig. 1C and Table 3). The postoperative 90 days mortality in the high-risk group was also significantly higher than that in the low-risk group (6.3% versus 1.3%, *P* = 0.045; Fig. 2C).

### Performance of ALBI-FIB4 score in predicting PHLF in subgroup analyses

The predictive ability of ALBI-FIB4 score for PHLF was further evaluated based on the extent of liver resection and tumor stage. In patients undergoing minor resection, the AUC of the ALBI-FIB4 score in predicting PHLF was 0.800 (95%*CI*: 0.688–0.911), higher than other models (Table 4). In patients undergoing major resection, the AUC of the ALBI-FIB4 score in predicting PHLF was also the highest (0.746, 95%*CI*: 0.578–0.913; Table 4). Consistently, the better accuracy of the ALBI-FIB4 score in predicting PHLF was also founded in both the early (BCLC-0 and A) and advanced (BCLC-B and C) HCC (Table 5).

**Table 4:** Performance of models in predicting PHLF in subgroups based on the extent of liver resection.

Models	Minor resection		Major resection	
	AUC	95%*CI*	AUC	95%*CI*
Child–Pugh score	0.583	0.445–0.720	0.615	0.423–0.808
MELD score	0.749	0.632–0.866	0.600	0.414–0.787
ALBI score	0.746	0.630–0.861	0.688	0.518–0.858
FIB-4 index	0.783	0.682–0.883	0.692	0.534–0.851
ALBI-FIB4	0.800	0.688–0.911	0.746	0.578–0.913

*ALBI* albumin-bilirubin, *FIB-4* fibrosis-4, *MELD* model for end-stage liver disease, *PHLF* post-hepatectomy liver failure.

**Table 5:** Performance of models in predicting PHLF in subgroups based on tumor stage.

Models	BCLC-0 and A stage		BCLC-B and C stage	
	AUC	95%*CI*	AUC	95%*CI*
Child–Pugh score	0.593	0.448–0.738	0.583	0.409–0.757
MELD score	0.629	0.485–0.773	0.745	0.656–0.834
ALBI score	0.754	0.645–0.863	0.707	0.546–0.867
FIB-4 index	0.774	0.663–0.885	0.726	0.599–0.853
ALBI-FIB4	0.802	0.695–0.909	0.766	0.613–0.919

*ALBI* albumin-bilirubin, *BCLC* barcelona clinic liver cancer, *FIB-4* fibrosis-4, *MELD* model for end-stage liver disease, *PHLF* post-hepatectomy liver failure.

## Discussion

The present study investigated the ability of the combination of ALBI score and FIB-4 index in predicting PHLF. Our results revealed that ALBI score and FIB-4 index were independent factors for predicting PHLF. The combined ALBI-FIB4 score showed greater ability in predicting PHLF, severe PHLF, and postoperative 90 days mortality than the commonly used liver function and fibrosis scoring systems including Child–Pugh score, MELD score, ALBI score, and FIB-4 index. The ALBI-FIB4 score could stratify patients into two groups of patients with significantly distinct risks of PHLF and postoperative 90 days mortality, which is helpful for the selection of appropriate patients for hepatectomy.

PHLF is a serious complication after HCC resection, which not only increases medical costs and prolongs hospital stay, but also may lead to postoperative death of patients^8,25^. In the current study, the incidence of PHLF was 16.3%. The postoperative 90 days mortality was 2.8%, and all died of PHLF. Our rates of PHLF and postoperative mortality were close to those reported in the literature^14,26–28^. To reduce the incidence of PHLF, patients with a single HCC and preserved liver function are considered ideal candidates for hepatectomy^2,3^. However, with the expansion of surgical indications, the prevalence of PHLF is still reported to be relatively high in the published literature and PHLF remains the leading cause of perioperative death in patients undergoing hepatectomy for HCC^8,9^. Thus, accurate preoperative estimation of the risk of PHLF is crucial to enhance the safety of hepatectomy for patients with HCC.

Compared with the traditional Child–Pugh score and MELD score, ALBI score has been proved to be a more reliable model for assessing liver reserve function in patients with HCC^13,17,29^. Wang and colleagues^13^ found that the ALBI score was more powerful than the Child–Pugh score in predicting PHLF and overall survival among patients who underwent liver resection for HCC. And the ALBI score could distinguish their patients with Child–Pugh grade A into two groups with distinct prognosis^13^. Fagenson and colleagues^17^ analyzed 13,783 patients undergoing hepatectomy, and their results showed that the ALBI score had better discrimination for PHLF and mortality compared with MELD score. Consistently, in this study, ALBI score also performed better than Child–Pugh score and MELD score in predicting PHLF and postoperative 90 days mortality. FIB-4 index has been extensively validated as a reliable and non-invasive marker of liver fibrosis^30^, and found to possess the capacity to forecast the clinical outcome of various chronic liver diseases, such as HBV, HCV, and NAFLD^31–33^. Moreover, the majority of patients with HCC are accompanied by chronic liver disease or even cirrhosis^3^. Around 80% of the patients in this study had HBV infection, and about 70% of them harbored liver cirrhosis. Accordingly, this combination is feasible in improving the accuracy of prediction of PHLF by looking at markers of liver function and fibrosis simultaneously.

This study performed multivariable regression analyses to identify preoperative factors associated with PHLF. And both the ALBI score and FIB-4 index were determined to be independent predictors of PHLF. Hence, we combined ALBI score with FIB-4 index to form a new scoring system, namely ALBI-FIB4 score. The ALBI-FIB4 score showed higher accuracy in predicting PHLF compared to the Child–Pugh score, MELD score, and either the ALBI score or FIB-4 score alone, although statistically significant difference based on the DeLong test was only achieved in comparison with the Child–Pugh score. This may due to the relatively small sample size of this study. Besides, the ALBI-FIB4 score also showed better ability in predicting postoperative 90 days mortality, with a high AUC of 0.843. These findings suggest that the ALBI-FIB4 score is a more accurate model for evaluating the safety of hepatectomy in HCC patients. The ALBI-FIB4 score consists of age and five biochemical indices including albumin, bilirubin, AST, ALT, and platelet count. These variables contained in the ALBI-FIB4 score are objective, non-invasive, and easily accessible, which is convenient for clinical application.

The ALBI-FIB4 score could divide patients into two risk groups based on an optimal cut-off value of − 1.82. High-risk patients had a high incidence of PHLF of 39.1%, while PHLF just occurred in 6.6% of low-risk patients. Furthermore, the postoperative 90 days mortality was up to 6.3% in the high-risk group, compared to the low rate of 1.3% in the low-risk group. Due to the high incidence of PHLF nearly 40.0% and 90 days mortality exceeding 5%, patients in high-risk group should be carefully selected for hepatectomy.

In addition to preoperative liver function, the scope of liver resection is another key factor affecting PHLF^4,8^. Consistent with previous reports, major liver resection was also identified as an independent risk factor of PHLF^13,28^. The risk of PHLF was 2.4 times higher in patients who underwent major hepatectomy compared with those who underwent minor hepatectomy. To examine the ability of the ALBI-FIB4 score to predict PHLF in patients undergoing different extent of liver resection, subgroup analyses were conducted. Our results showed that the ALBI-FIB4 score displayed greater accuracy than other models in predicting PHLF in both patients undergoing minor and major hepatectomy. In addition, in both the early (BCLC-0 and A) and advanced (BCLC-B and C) HCC patients, the ALBI-FIB4 score also showed better accuracy in predicting PHLF.

This study had several limitations. First, most patients in the current study were HBV-related HCC, unlike in many Western countries where HCV infection and NAFLD are the main causes of HCC. Thus, the results of this study need to be further confirmed from HCC patients with different etiologies. Second, the study was conducted retrospectively, meaning that it relied on past data and records, which can introduce certain biases into the study results. Another limitation of this study is that it was conducted at a single medical center, with a relatively small sample size. The predictive ability of the ALBI-FIB4 score established in this study for PHLF needs further external validation.

## Conclusions

ALBI-FIB4 score showed good performance in predicting PHLF, severe PHLF, and postoperative mortality in patients undergoing hepatectomy for HCC, superior to the currently commonly used liver function and fibrosis scoring systems including Child–Pugh score, MELD score, ALBI score, and FIB-4 index.

## Data Availability

All data analyzed during this study are included in this published article.
